# Nucleocytoplasmic Shuttling of STATs. A Target for Intervention?

**DOI:** 10.3390/cancers11111815

**Published:** 2019-11-19

**Authors:** Sabrina Ernst, Gerhard Müller-Newen

**Affiliations:** 1 Institute of Biochemistry and Molecular Biology, RWTH Aachen University, 52074 Aachen, Germany; sabrina.ernst@rwth-aachen.de; 2 Confocal Microscopy Facility, Interdisciplinary Center for Clinical Research IZKF, RWTH Aachen University, 52074 Aachen, Germany

**Keywords:** STAT3, STAT5, nuclear pore complex, nuclear transport receptors, nucleocytoplasmic shuttling, cancer, targeting

## Abstract

Signal transducer and activator of transcription (STAT) proteins are transcription factors that in the latent state are located predominantly in the cytoplasm. Activation of STATs through phosphorylation of a single tyrosine residue results in nuclear translocation. The requirement of tyrosine phosphorylation for nuclear accumulation is shared by all STAT family members but mechanisms of nuclear translocation vary between different STATs. These differences offer opportunities for specific intervention. To achieve this, the molecular mechanisms of nucleocytoplasmic shuttling of STATs need to be understood in more detail. In this review we will give an overview on the various aspects of nucleocytoplasmic shuttling of latent and activated STATs with a special focus on STAT3 and STAT5. Potential targets for cancer treatment will be identified and discussed.

## 1. Aim and Scope

STAT (Signal transducer and activator of transcription) proteins can be seen as intracellular messengers that relay signals sensed at the plasma membrane to chromatin and genes in the nucleus. To achieve this, STATs must pass the nuclear envelope through nuclear pore complexes (NPCs). Thus, passage through the NPC is an essential step in the sequence of events from activation of STATs at cytokine receptors to DNA-binding and target gene induction. As detailed in the reviews and articles of this Special Issue of Cancers, deregulated activation of STAT3 and STAT5 contributes to various cancers in many ways. Thus, STAT3 and STAT5 proteins have emerged as promising therapeutic targets. Protein–protein interactions involved in nucleocytoplasmic transfer of STATs have not been exploited yet as molecular targets for intervention. Detailed knowledge of the involved molecules and mechanisms is an essential prerequisite for successful and specific targeting. In this review, we will first describe the general mechanisms involved in import and export of proteins in and out of the nucleus, concentrating on those which are most relevant for transcription factors. We will then focus on the mechanisms involved in nucleocytoplasmic shuttling of STAT3 and STAT5 and finally assess possible molecular targets for specific intervention.

## 2. General Mechanisms of Nucleocytoplasmic Transport of Proteins

### 2.1. The Nuclear Pore Complex

To enter or exit the nucleus, proteins must pass through the nuclear pore complex (NPC) [1]. NPCs are huge macromolecular assemblies (about 120 MDa in humans) made up of multiple copies of Nucleoporins (NUPs). More than 30 different NUPs have been identified that are built into the NPC as multiples of eight (8–64) resulting in the eight-fold rotational symmetry of the NPC [2]. The NPC can be seen as a channel that allows selective transfer of cargo and at the same time forms a soft barrier for free diffusion of macromolecules larger than about 30 kDa [3,4], preventing their access to the nucleus without permission. The barrier is formed by phenylalanine-glycine (FG)-repeats that protrude from certain NUPs into the lumen of the channel [5,6].

How exactly the FG-repeats form a selective permeability barrier is not completely understood and several models are currently being discussed [7,8]. One of the most prevalent is the selective phase model that relies on interactions between the FG-repeats creating a sieve-like meshwork with hydrogel-like properties, which would explain the observed mass exclusion limit [9]. Selectivity for cargo allowed to pass might result from phase separation that prevents passage of macromolecules that are unable to mix or interact with the selective phase made up by the FG-repeats [8].

The import/export pathways through the NPC involve soluble nuclear transport receptors (NTRs) that bind cargo in conjunction with the Ran-GTP/GDP cycle. NTRs can interact with FG-repeats [10] and facilitate passage of bound cargo through the NPC. According to the selective phase model, interaction of NTRs with the FG-repeats leads to local disturbance of the meshwork allowing the NTR/cargo complex to travel almost freely between cytoplasm and nucleoplasm [11]. The energy-consuming Ran-GTP/GDP cycle provides directionality of the transport through control of cargo to NTR binding which is differently regulated in nucleoplasm vs. cytoplasm [12,13].

NTRs, also known as Karyopherins, can be subdivided in Importins and Exportins, facilitating nuclear import and export, respectively. Biportins have also been described that support transport of cargo in both directions with imported and exported cargo being distinct [14].

### 2.2. Importins

The best characterized Karyopherin is Importin-β1 which either binds cargo directly or indirectly through interaction with adapters such as α-Importins or Snurportin-1 [15]. The transcription factors Snail1 [16,17] and SREBP2 [18] are among the cargoes directly bound by Importin-β1. Snurportin-1 is best known for its involvement in the nuclear import of spliceosomal snRNPs [19]. The interaction site of Importin-β1 with FG-repeats has been mapped [20,21] and is different from the well-defined interaction sites with cargoes and adapters [22]. This means that cargo-loaded Importin-β1 can interact with FG-Nucleoporins of the NPC and thereby facilitate passage of the Importin-β1/adapter/cargo complex.

The import pathway using α-Importins as adapters to connect cargo with Importin-β1 has been intensively studied and is now known as the classical import pathway [23]. Accordingly, the term classical nuclear localization signal (cNLS) refers to linear sequence motifs of cargoes that bind to α-Importins. The cNLS can be further subdivided into monopartite cNLS and bipartite cNLS. Monopartite cNLS consist of a short stretch of basic amino acid residues, the sequence PKKKRRV of the SV40 large T-antigen being the first identified [24]. The first bipartite cNLS has been found in the Nucleoplasmin protein of Xenopus laevis, consisting of two short stretches of basic amino acids, both essential for its function, separated by a few less relevant amino acids [25].

In humans, seven Importin-α isoforms have been identified: Importin-α1, -α3, -α4, -α5, -α6, -α7 [23] and the most recently discovered Importin-α8 [26]. All α-Importins are mainly built up of ten Armadillo (ARM)-repeats resulting in a structurally conserved solenoid protein domain [27]. The cNLS of cargo binds along a groove on the inner concave surface of α-Importins. Although the cNLS binding region is quite conserved, the α-Importins are specific for a set of cargo proteins with some considerable overlap. This specificity results in part from preferential binding of certain NLS but also from the three-dimensional context in which the NLS is presented by the cargo [28]. Apart from cNLS, so called non-classical or atypical NLS have been identified, which do not fit in the cNLS consensus motifs [29]. A short sequence termed Importin- β binding (IBB) domain precedes the solenoid domain of α-Importins. In the absence of cargo, the IBB folds back to the NLS binding site. Upon binding of cargo, the IBB is replaced by the cNLS of the cargo protein. The now exposed IBB binds to Importin-β1, facilitating the nuclear import of the ternary Importin-β1/Importin-α/cargo complex [30].

Transportin-1, also known as Importin-β2, also imports a broad spectrum of cargoes, including transcription factors and mRNA-binding proteins. Transportin-1 binds cargo directly through a broad range of loosely related NLS that are different from cNLS [31]. Symportin-1 has been identified as an adapter for nuclear import of some ribosomal proteins by Transportin-1 [32]. Transportin-3 recognizes arginine-serine (RS)-rich NLS found in proteins typically involved in mRNA metabolism such as the Splicing Factor 2 (SF2) [33]. Importin-13 is closely related to Transportin-3 but mediates both protein import and export. Therefore, by definition Importin-13 can be regarded as a Biportin. Some transcription factors are among the many cargoes identified so far. Cargo binding by Importin-13 relies on the recognition of folded domains rather than linear NLS [34,35].

Some import pathways have been identified that do not rely on β-Importins. Among those, Calmodulin-mediated nuclear import seems to be most relevant for transcription factors [36]. Finally, some proteins including transcription factors enter the nucleus independent of NTR or other carrier proteins, e.g., through direct interaction with NUPs [37].

### 2.3. Exportins

Crm1 (Chromosome region maintenance 1; in the systematic nomenclature of Karyopherins designated as Exportin-1 or Xpo1) is the most promiscuous Exportin, which mediates the export of about 1000 substrates, including various RNAs, ribonucleoproteins, and transcription factors [38]. Most protein cargo of Crm1 contains a leucine-rich nuclear export signal (NES) of 8–15 amino acids [39,40,41]. Like all β-Karyopherins, Crm1 is built up of HEAT-repeats (Huntingtin, Elongation factor 3, protein phosphatase 2A and TOR kinase). Each HEAT-repeat consists of two α-helices connected by loops of varying length. Similar to the ARM repeats of α-Importins, the HEAT-repeats of β-Karyopherins form a solenoid protein domain [42] with a slightly curved superhelical structure of high conformational flexibility. In all of the available structures of cargo bound to Crm1, the NES of the cargo fits into a hydrophobic groove between HEAT repeats 11 and 12 (out of 20) at the outer convex surface of Crm1 [43]. Binding at the outer convex surface of Crm1 allows accommodation of cargo of various sizes.

Other Exportins, such as Exportin-5, Exportin-t, and Exportin-2 (better known as CAS for Cellular Apoptosis Susceptibility), bind their cargo on the inner concave surface. Accordingly, these Exportins are more selective. Exportin-5 mediates export of several types of RNA but not protein; Exportin-t is even more restricted to the export of tRNAs [44]. CAS is specialized for the nuclear export of α-Importins, which is needed to maintain the classical import pathway [45]. Exportin-4, a distant member of the β-Karyopherin family, originally described to mediate nuclear export of the translation initiation factor 5A [46] and the transcription factor Smad3 [47], has more recently been described to be involved in the nuclear import of Sox transcription factors [48], thus acting as a Biportin. Another Biportin, Exportin-7 (also known as RanBP16) [49], has recently been described to act as a broad-spectrum Karyopherin with about 200 export and 30 import substrates [50].

### 2.4. Ran-GTP/GDP Cycle

In principle, through interaction with the FG-repeats, β-Karyopherins can pass the NPC in both directions [51]. Directionality of nucleocytoplasmic transport is achieved through coupling of cargo binding to β-Karyopherins with the energy-consuming Ran-GTP/GDP cycle [12,52]. Ran is a small G-protein of the Ras superfamily of GTPases that exists in a GTP- or GDP-bound state. The GTP-bound state results from replacement of bound GDP with GTP that requires the activity of a Ran-specific, chromatin-bound guanine-nucleotide exchange factor (RanGEF, also known as RCC1), which is predominantly located in the nucleus. Therefore, the Ran-GTP concentration in the nucleus is high. The GDP-bound state is generated through release of the β-phosphate of bound GTP by hydrolysis, which requires the activity of a ran-specific GTPase-activating protein (RanGAP). Through interaction with the Nucleoporin Ran-binding protein 2 (RanBP2, Nup358), RanGAP is located predominantly at the cytoplasmic surface of the NPC. Therefore, in the cytoplasm close to the nuclear envelope, the Ran-GTP concentration is low and the Ran-GDP concentration is high [53,54].

Cargo binding to Importins in the cytoplasm is Ran-independent. After passage of the Importin/cargo complex through the NPC, release of cargo in the nucleus is induced upon Ran-GTP binding. In contrast, Ran-GTP-binding to Exportins is required for cargo loading in the nucleus. After passage of the Exportin/cargo/Ran-GTP complex through the NPC, RanGAP-mediated GTP-hydrolysis results in release of RanGDP and cargo into the cytoplasm [55].

As mentioned above, NTRs facilitate nucleocytoplasmic transport through transient interactions with the hydrophobic FG-repeats within the channel of the NPC. In principle, other proteins with hydrophobic surface patches should to some extent also be able to disturb the FG-repeat meshwork, allowing them to shuttle between the cytoplasm and the nucleus in an NTR- and Ran-independent, passive manner. Indeed, in a recent study [56] it has been found that a continuum exists with gradual differences in the ability of proteins to pass the NPC in an active (i.e., NTR- and Ran-dependent) or passive manner. Depending on their surface properties, even large proteins can leak into the nucleus without involvement of NTRs.

## 3. STAT1—Using a Side Track for Nuclear Import

**Figure 1:**
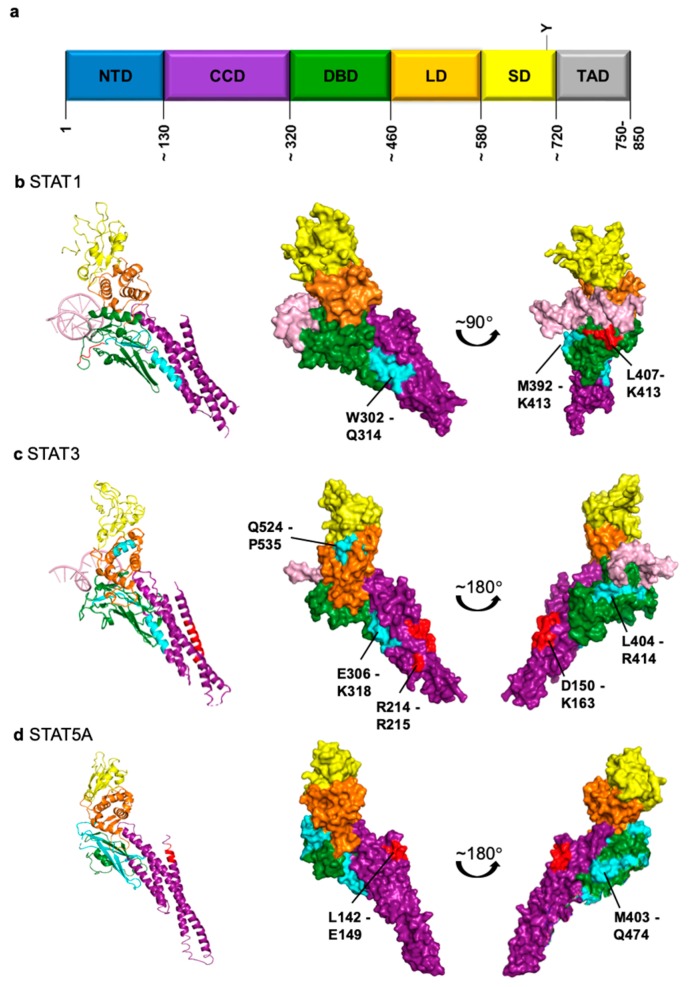
Structures of STAT proteins with putative nuclear localization signals (NLS) and nuclear export signals (NES) highlighted as listed in Table 1. In this context, “putative” means that the corresponding sequences do not fulfill classical NLS or NES functions but are required for nuclear transport or interaction with nuclear transport receptors. (**a**) General scheme of structural domains of STAT proteins (NTD, N-terminal domain; CCD coiled-coil domain; DBD, DNA-binding domain; LD, linker domain; SD, SH2 domain; TAD, transactivation domain). Numbers refer to amino acid positions. (**b**–**d**) Structures of individual STAT proteins as ribbon representations (left) or space-filling representations (middle and right). Domains are stained according to the coloring of the scheme in (**a**), DNA is shown in pink. Putative NLS and NES are marked in red and cyan, respectively. The corresponding references are listed in Table 1. PDB IDs: STAT1, 1BF5; STAT3, 1BG1; STAT5A, 1Y1U. Images of structures were generated with PyMOL.

STAT1 is deeply involved in the antiviral response triggered by endogenously produced Interferons but also in the therapeutic responses to exogenously administered Interferons in cancer treatments and anti-viral therapies [57]. STAT1 is best known for its tumor suppressive activity in cancer but some tumor-promoting effects have also been documented [58]. IFNγ (type II Interferon) induces the activation of STAT1 through phosphorylation of Y701 by IFNγ receptor-associated Janus kinase 1 (JAK1) and JAK2, leading to dimerization and nuclear accumulation of the transcription factor [59]. Among all STAT family members, the mechanism of nuclear accumulation of the activated STAT1 dimer is best understood. Activated STAT1 interacts with Importin-α5 [60], but in an unconventional manner. Instead of employing the binding site for cNLS, which would involve ARM repeats 2–4 and 6–8 as the major and minor binding sites, respectively [22], STAT1 binds to the more C-terminally located ARM repeats of Importin-α5 [61] involving a critical tyrosine residue (Y476) located in ARM repeat 10 [62]. Accordingly, the activated STAT1 dimer does not expose a cNLS but a dimer-specific surface area to interact with Importin-α5. This surface has been termed dimer-specific NLS (dsNLS) and includes regions within the N-terminal domain (NTD) [63] and the DNA-binding domain (DBD; see Figure 1a for a scheme of the domain structure of STAT proteins; see Table 1 for an overview on putative NLS and NES sequences of STAT proteins; and see Figure 1b showing the corresponding putative NLS and NES motifs in the STAT1 dimer structure) [64]. Hence, Importin-α5 can be displaced from STAT1 by DNA-binding [65,66]. Most interestingly, this unique mode of interaction is exploited by the Ebola virus VP24 protein, that interacts with ARM repeats 8–10 of Importin-α5, preventing binding and nuclear translocation of activated STAT1 [67]. Interaction of Importin-α5 with cNLS cargo remains unaffected by VP24 binding. This example shows that, in principle, nuclear accumulation of a STAT transcription factor can be selectively blocked.

## 4. STAT3—Acting on Many Stages

### 4.1. Role of STAT3 in Cancer

STAT3 is involved in cancer in multiple ways, as detailed in several reviews and articles of this Special Issue of Cancers. Besides acting as an oncogene in a cell autonomous manner [80,81], activation of STAT3 in the tumor microenvironment contributes to metastasis, angiogenesis, cancer stem cell maintenance, and immune evasion [82]. Therefore, blocking STAT3 activity in cancer is of particular interest. However, it should also be noted that in some cancers STAT3 has tumor suppressive activities [83,84]. Several steps in the STAT3 activation pathway are potentially targetable and thus might be used to interfere with STAT3 activation. These include phosphorylation on Y705 by (oncogenic) tyrosine kinases, dimerization through phosphotyrosine/SH2 domain interactions, nuclear translocation, DNA-binding to GAS (γ-interferon activated sequence) elements, and interaction with cofactors. However, passage of the activated STAT3 dimer through the NPC has only rarely been considered as a potential molecular target for intervention [85,86]. This might be due to the fact that import mechanism for activated STAT3 and the interactions of STAT3 with NTRs have been analyzed in some detail but are not completely understood as outlined below.

### 4.2. Nuclear Import

Compared to STAT1, the mechanism of nuclear import of the activated STAT3 dimer is less well defined. Reports on interactions with α-Importins are contradictory in part. In one of the first studies, interactions of STAT3 with Importin-α1, -α3, and -α5 were detected [70]. Additionally, interactions of STAT3 with Importin-α5 and -α7, but not with Importin-α1, -α3, and -α4, were found [69]. In both reports, interactions were only seen upon activation of STAT3. Other groups found interactions with Importin-α3 and -α6 [71] and Importin-α3, -α5, and -α7 [72] being independent of activation of STAT3. In this context it is worth noting that expression of Importin-α6 is restricted to testis [87]. Therefore, Importin-α6 is less relevant for nuclear import of ubiquitously expressed transcription factors such as STAT3 and STAT5. Based on import assays with permeabilized cells, the MgcRacGAP protein bound to Rac has been suggested as an NLS-containing adapter for Importin-α-mediated nuclear import of STAT3 and STAT5 [88]. In another study using an MgcRacGAP inhibitor and siRNA mediated knockdown, the functional role of MgcRacGAP for nuclear import of STAT3 could not be substantiated [89]. These discrepancies might be the result of different experimental conditions and cellular systems used. For instance, subcellular distribution of STAT3 analyzed by immunofluorescence heavily depends on the applied fixation method [90]. Alternatively, the different findings indicate that STAT3 employs a different import system depending on the cytokines used for its activation, e.g., Interleukin-6 (IL-6) vs. Epidermal Growth Factor (EGF). Clearly, more work is required for better understanding.

Functional relevance of Importin-α5 in nuclear import of activated STAT3 has been demonstrated, as well as the involvement of Importin-β and Ran [70]. Moreover, it has been shown that the epitope on Importin-α5 employed in the interaction with STAT3 is different from the one used for the interaction with STAT1. A mutation in ARM repeat 10 of Importin-α5 blocks interaction with STAT1 [62] but does not affect the interaction with STAT3 [72]. Correspondingly, the epitopes used by STAT3 and STAT1 for interaction with Importin-α5 are also different. Amino acids in the DBD of STAT1 are involved in the recognition of Importin-α5 [65,66], connecting Importin-binding and nuclear presence with DNA-binding activity of STAT1 [91]. In STAT3, mutation of R214 and R215 located in the coiled-coil domain (CCD) abolishes binding of Importin-α5 and nuclear accumulation in response to stimulation [69]. Liu et al. [71] defined the sequence D150-K163 as being indispensable for nuclear import of STAT3, which is also located in the CCD. Nuclear accumulation of activated STAT3 does not require DNA-binding activity [72]. Common to STAT3 and STAT1 is the involvement of the N-terminal domain in stimulation-dependent nuclear import [63,74,75]. The essential role of the NTD for active nuclear import of STAT3 is further supported by the observation that the tumor suppressor ARHI (A Ras Homologue member I/DIRAS3) blocks nuclear translocation of phosphorylated STAT3 via direct interaction with the NTD [92].

It seems that Importin-α5 is a major determinant for nuclear import of STAT3 and STAT1 but the molecular interfaces of the interactions are considerably different. This opens an avenue for specific interventions. However, to achieve a selective block of nuclear import of STAT3, the structural details of the interactions with NTRs must be characterized in more detail. It should be noted that no classical NLS or NES sequences have been identified in STAT3 in the sense of transferable functional motifs. The above-mentioned amino acids in the putative NLS and NES are most probably important for the integrity of domain structures or the part of epitopes that interact with NTRs in a non-conventional manner [75], as exemplified for STAT1 [62] (Figure 1c showing putative NLS and NES motifs in the activated STAT3 dimer structure).

### 4.3. Nuclear Export and Nucleocytoplasmic Shuttling

In the course of defining nuclear export signals of STAT3, it was found that pharmacological inhibition of Crm1-mediated nuclear export results in partial nuclear accumulation of STAT3 independent of cytokine stimulation [79]. From these and other studies it became evident that the subcellular distribution of latent STAT3 with high cytoplasmic and low nuclear concentration is the result of a steady-state of constitutive nuclear import and rapid export independent of phosphorylation at Y705 [93,94]. Determinants of stimulation-dependent nuclear import, such as the NTD and the R214/R215-motif, are not involved in basal nuclear import of latent STAT3 [75]. Unphosphorylated STAT1 has also been detected in the nucleus [95], and the mechanisms of nuclear import of latent and activated STAT1 were also found to be different [64]. Nuclear import of unphosphorylated STAT3 might be independent of NTRs and could involve direct interactions with FG-repeat NUPs of the NPC as observed for STAT1 [96]. From the three sequence motifs identified as putative NES in STAT3, at least the one containing L525/L528 is involved in constitutive nucleocytoplasmic shuttling [75,79].

It has been firmly established that STAT3 and STAT1 form homodimers in the absence of the activating tyrosine phosphorylation resulting in so called preformed dimers [97,98]. The phosphotyrosine-independent dimerization involves homotypic interactions of the NTDs [99]. However, basal nucleocytoplasmic shuttling of STAT3 does not require the NTD [75] and is not sensitive to a single point mutation (L78R) that prevents preformed dimer formation [100]. Not only is dimer formation not required for basal shuttling, but monomeric STAT3 shuttles even faster [75]. The increased shuttling rate of monomeric STAT3 could be attributed to the smaller size compared to the preformed dimer. However, a recent study suggests that hydrophobic surface patches that interact with FG-repeats of FG-Nups might be a stronger predictor for NTR-independent passage of the NPC than size [56]. Thus, accessible hydrophobic surfaces that are masked in the dimer may facilitate faster shuttling of monomeric STAT3 in an NTR-independent manner.

Since basal and stimulation-dependent nuclear import of STATs occur through different mechanisms, they could, in principle, be targeted independently of each other. However, the functional relevance of nuclear presence of unphosphorylated STAT3 and STAT1 (U-STATs) is not entirely clear. Nuclear U-STATs might be involved as cofactors in gene regulation independent of their DNA-binding activity [101].

## 5. STAT5—Leukemia and More

Important functional roles in hematopoiesis downstream of hematopoietic cytokines such as Erythropoietin and Thrombopoietin have been attributed to STAT5. Consequently, deregulated STAT5 signaling is prominently implicated in myeloproliferative diseases and leukemias [102]. The involvement of STAT5 in solid tumors is also well documented, both as an oncogene and a tumor suppressor [103], as detailed in several reviews and articles of this Special Issue of Cancers. In particular, in myeloproliferative neoplasms and leukemias, STAT5 has been identified as a promising therapeutic target.

Compared with the many reports on STAT3 and STAT1, only a handful of studies dedicated to the molecular mechanisms governing nucleocytoplasmic distribution of STAT5 exist. In one of the first studies [104], a sequence motif in the DNA-binding domain of STAT5B (V466-I469) was identified whose mutation prevented Growth Hormone-induced nuclear accumulation and DNA-binding. However, from this finding it cannot be concluded that DNA-binding per se is required for nuclear accumulation of activated STAT5B because the mutation could also interfere with binding of NTRs, which was not investigated in this study. We found that another mutation that affects DNA-binding of STAT5A does not impair nuclear accumulation upon Epo stimulation, in agreement with the DNA-binding independent nuclear accumulation of activated STAT3 [72]. In general, nuclear accumulation of STATs through retention by binding to nuclear structures seems to be of minor importance. If nuclear accumulation was dependent on retention of STATs on subnuclear structures, nuclear accumulation would be saturable, meaning that as soon as all binding sites are occupied, the remaining STAT molecules would not accumulate in the nucleus. This is not what is observed in the above-mentioned experiments with transfected cells: Even upon forced overexpression, STATs almost completely accumulate in the nucleus upon stimulation. This view is supported by the results of fluorescence recovery after photobleaching (FRAP) experiments that have been exemplarily performed on STAT1-GFP. Upon nuclear accumulation induced by Interferon treatment, STAT1-GFP freely diffuses through the nucleoplasm with the exclusion of nucleoli [105]. In this and another study [93], the involvement of the cytoskeleton in directed nuclear import of STAT1 and STAT3 was also excluded.

In one of the reports on binding of α-Importins to STAT3, binding to STAT5A and STAT5B was analyzed in parallel but no interaction could be detected [69]. This is in agreement with our own observations made with co-precipitation experiments from cellular lysates using Importins fused to GST (glutathione-S-transferase). Accordingly, no cNLS has been detected in STAT5. In another study, interaction of STAT5A with Importin-α3 has been shown [73], again supporting the notion that the outcomes of in vitro assays (mostly co-precipitations) for studying the interaction of STATs with NTRs are very sensitive to the experimental conditions used.

Similar to STAT3 and STAT1, constitutive nucleocytoplasmic shuttling of STAT5 independent of tyrosine phosphorylation has been detected [77]. A functional role of nuclear U-STAT5 has been established in megakaryocyte differentiation [106]. Basal and cytokine-induced import of STAT5A requires an intact CCD [73] and was abolished upon deletion of eight amino acids in the CCD (L142–E149, see Table 1 and Figure 1d showing putative NLS and NES motifs in the STAT5A monomer structure; a structure of the activated STAT5 dimer is not available) [76]. Interaction of STAT5A with Importin-α3 occurs in an unconventional manner involving the CCD [73]. As for STAT3 and STAT1, stimulation-dependent but not basal nuclear translocation requires the NTD of STAT5 [77]. In a study that tested all sequence motifs that might function as a cNES, a region between aa578 and aa675 in STAT5B comprising the SH2 domain was identified to be required for nuclear export [77]. Another region important for Crm1-mediated export was identified involving L119 and L133 in the NTD of STAT5A [73].

A discrepancy between nuclear localization of STAT5A and STAT5B was found in response to phosphorylation of the critical tyrosine residue (Y694 in STAT5A and Y699 in STAT5B) by Src family kinases (SFK). While phosphorylation of Y699 in STAT5B, as well as Y705 in STAT3, by Src leads to nuclear accumulation of the transcription factors, phosphorylation of Y694 in STAT5A by Src does not [107]. Later it was found that this observation is of some relevance in Bcr-Abl-positive chronic myeloid leukemia (CML), where STAT5 is predominantly localized in the cytoplasm despite being phosphorylated at the critical tyrosine residue [108]. Cytoplasmic retention of STAT5A in response to activation by Bcr-Abl was found to be mediated by SFK such as Src and Hck. Specific inhibition of SFKs resulted in nuclear accumulation of STAT5A, enhanced STAT5 target gene expression, and increased colony formation of CML cells [109]. SFK interfere specifically with dimerization of activated STAT5A and thereby prevent nuclear accumulation [110]. In another report, phosphorylation of S779 has been suggested as an additional requirement for the nuclear translocation of STAT5A in Bcr-Abl positive cells [111]. These intriguing examples show that even the highly homologous transcription factors STAT5A and STAT5B differ in some aspects of nucleocytoplasmic shuttling and these differences might be relevant for disease.

## 6. Perspectives for Therapeutic Interventions

Nucleocytoplasmic transport has been recognized as a target for cancer therapy [112,113] in part based on the observation that components of the nuclear transport machinery are differentially expressed in transformed cells [114]. These changes in expression can be relevant for disease. It has been shown that Crm1-mediated nuclear export contributes to drug resistance in multiple ways [115]. For example, Topoisomerase II inhibitors such as doxorubicin require nuclear Topoisomerase activity. As a drug resistance mechanism, Topoisomerase II can be exported from the nucleus in a Crm1-dependent manner [116]. Treatment with Crm1 inhibitors therefore sensitizes cancer cells to doxorubicin treatment [117]. Only recently, in two independent studies, was Crm1 convincingly identified as a synthetic lethality gene in different types of cancer [118,119]. Earlier clinical studies already showed that the highly efficient Crm1 inhibitor leptomycin B is not well tolerated by patients [120]. Less toxic small molecule inhibitors of Crm1 termed Selective Inhibitors of Nuclear Export (SINE), such as selinexor, are currently used in clinical trials in patients with hematological malignancies and solid tumors [112]. Most recently, selinexor in combination with dexamethasone has been approved for the treatment of refractory multiple myeloma. In this context, it has already been recognized that a more selective export inhibition could be more effective and reduce the serious side effects of treatment [115]. There are only a few inhibitors for some other NTRs available (Importin-β1, Importin-α/β1 heterodimers, and Transportin-1) which have not been explored in such detail yet [121].

For transcription factors that respond to extracellular cues, control of nucleocytoplasmic distribution contributes to efficient signal transduction. High cytoplasmic concentration of the latent transcription factor increases the sensitivity to stimulation, which usually occurs at the cytoplasmic face of membrane-bound receptors. After stimulation and activation of the transcription factor, high nuclear concentration increases the efficiency of DNA-binding and thus gene induction. However, the molecular mechanisms involved in the control of subcellular distribution vary considerably between different transcription factors. For instance, the NLS of NF-κB is masked by IκB [122]. Upon sensing of inflammatory mediators by membrane-bound receptors, IκB is phosphorylated and ubiquitinated resulting in its proteasomal degradation. The now exposed NLS facilitates α-Importin-mediated nuclear accumulation of NF-κB [123]. Likewise, the NLS of some nuclear receptors, such as the Glucocorticoid Receptor and Estrogen Receptor, are masked. Ligand binding to the receptors results in release of Hsp90 and exposition of the NLS [124]. Similar cytoplasmic interaction partners that would prevent nuclear translocation of latent STAT proteins have not been identified yet. Various other mechanisms involving SUMOylation, ubiquitination, mono-ADP-ribosylation, and acetylation have been described to control the nucleocytoplasmic distribution of transcription factors [22,125]. Beside the well-established Karyopherin-mediated transport, other non-conventional transport mechanisms exist [126]. Nucleocytoplasmic transport of β-Catenin is regulated independently of Ran and classical transport factors [127,128]. At the same time, β-Catenin acts as a transport receptor for its transcription factor partner LEF-1 [129]. The regulatory 14-3-3 proteins have also been suggested to be involved in such a piggy-back mechanism of nuclear transport [130,131], which has so far not been described for STAT proteins. Because of these various transport modes, specific interference should be feasible in principle.

In the case of STAT transcription factors, a non-classical NLS is generated through phosphorylation-induced dimerization (dsNLS) that is recognized by α-Importins or other yet uncharacterized factors. Because this mechanism is quite unique, a specific intervention through targeting the dsNLS/α-Importin interface should in general be feasible, as exemplified for STAT1 [67]. Extended surface areas are involved in the STAT/NTR interaction, including the NTD, DBD and CCD. The NTD seems to be a common denominator of nuclear import of activated STATs. Because the NTDs of different STATs vary in their sequence, a specific targeting should be possible, as exemplified by the specific interaction of ARHI with the NTD of STAT3, but not with the NTDs of STAT5 and STAT1 [92]. The impact of the ARHI/STAT3-NTD interaction on Importin-binding was further analyzed in a study applying homology modelling and molecular dynamics simulation [132]. Indeed, targeting of the NTD of STAT3 has been reported, however, with a focus on the role of the NTD as a tetramerization domain at enhancers and its function in gene induction [133]. The more recently solved structure of the NTD of STAT3 [134] should pave the way for a closer inspection of the surface areas involved in nuclear import by mutation of surface-exposed residues. For nuclear import of STAT5 and STAT3, data concerning use of the classical α-Importin/β1-Importin pathway are conflicting. Here, a fresh approach is needed to identify the NTRs involved, including α-Importins/Importin-β1-independent pathways mentioned in Section 2 of this review.

Reduced export of the imported STAT dimers also contributes to nuclear accumulation because export and redistribution requires dephosphorylation [135]. Blocking nuclear export of STATs as a therapeutic approach seems at first sight counterintuitive because it might trap the activated transcription factor in the nucleus where it could drive persistent oncogenic gene induction. However, STAT1, STAT3, and STAT5 are dephosphorylated in the nucleus by TC45, the 45 kDa isoform of T Cell Protein Tyrosine Phosphatase (TC-PTP) [136,137,138]. For STAT1 it has been shown that only the dephosphorylated form leaves the nucleus for reactivation at the receptor [91]. Exit out of the nucleus is required for reactivation of STATs [139]. Thus, STAT-mediated gene induction could be inhibited by trapping the dephosphorylated, transcriptionally inactive STATs (in the sense of canonical STAT signaling) in the nucleus through blockade of nuclear export. This has already been shown for STAT3 using the Crm1 inhibitor ratjadone A [140]. The more clinically advanced SINE have also been tested. These compounds inhibited STAT3-mediated Survivin expression in breast cancer models [141]. To interfere more specifically with export of STATs, the Exportins or Biportins involved must be characterized and the molecular interfaces of the STAT/NTR interaction mapped in detail.

Mechanistically, the export of dephosphorylated STATs most probably corresponds to the exit mechanisms involved in constitutive shuttling of latent STATs. Treatment of cells with specific inhibitors for the Exportin Crm1 often leads to only a partial nuclear accumulation of latent STATs. This means that either passive NTR-independent export occurs through direct hydrophobic interactions with FG-Nups or that other Exportins are involved. Specifically for STAT5A, a Crm1-independent export signal in the DBD has been mapped [73]. Redundancy in cargo recognition by NTRs in the sense that one cargo is transported by several NTRs is not unusual. As outlined in Section 2, broad-specificity Exportins or Biportins have been identified in recent years, worthy of analysis with respect to their involvement in the nuclear export of STAT proteins.

## 7. Conclusions

Taken together, effective inhibition of oncogenic STAT activity can in principle be achieved through specific blockade of both nuclear import of phosphorylated STAT dimers and export of dephosphorylated STATs out of the nucleus. However, this requires a deeper understanding of the protein–protein interactions involved in these processes. First, all NTRs facilitating nucleocytoplasmic shuttling need to be unambiguously identified for the individual STAT proteins. These studies should also include those NTRs that have been characterized in more detail in recent years. Then, the interactions between STATs and NTRs must be mapped in more detail using all structural data available. The ultimate goal would be to solve the structure of STAT/NTR complexes by X-ray crystallography or cryo-electron microscopy. Furthermore, the contribution of NTR-independent nuclear transport of STATs should be evaluated through assessment and mutation of surface patches as delineated by Frey et al. [56]. Based on solid structural and functional data, specific blockade of nucleocytoplasmic transport of individual STATs by tailor-made inhibitory molecules might be feasible.

## Figures and Tables

**Table 1:** NTRs, NLS and NES involved in transport of STAT proteins.

	STAT1	STAT3	STAT5A	STAT5B
Importin	α5 [60,68], α7 [69]	α1 [70], α3 [70,71,72], α5 [69,70,72], α6 [71], α7 [69,72]	α3 [73]	n.d. ^1^
Putative ^2^ NLS	L407-K413 [64,68] NTD involved [63,74]	D150-K163 [71] R214/R215 [69] NTD involved [75]	L142-E149 [76] intact CCD required [73]	n.d.
Exportin	Crm1 [66]	Crm1 [75]	Crm1 (not exclusively) [73]	Crm1 (not exclusively) [77]
Putative ^2^ NES	W302-Q314 [78] M392-K413 [66]	E306-K318 L404-R414 Q524-P535 [79]	L119/L133 M403-Q474 [73]	n.d.

^1^ n.d., not determined; ^2^ in this context, “putative” means that the corresponding sequences do not fulfill classical NLS or NES functions but are required for nuclear transport or interaction with NTRs.
